# Modeling the predictive potential of extralinguistic context with script knowledge: The case of fragments

**DOI:** 10.1371/journal.pone.0246255

**Published:** 2021-02-11

**Authors:** Robin Lemke, Lisa Schäfer, Ingo Reich

**Affiliations:** 1 Collaborative Research Center 1102, Project B3, Saarland University, Saarbrücken, Germany; 2 Department of German Studies, Modern German Linguistics, Saarland University, Saarbrücken, Germany; Northumbria University, UNITED KINGDOM

## Abstract

We describe a novel approach to estimating the predictability of utterances given extralinguistic context in psycholinguistic research. Predictability effects on language production and comprehension are widely attested, but so far predictability has mostly been manipulated through local linguistic context, which is captured with *n*-gram language models. However, this method does not allow to investigate predictability effects driven by extralinguistic context. Modeling effects of extralinguistic context is particularly relevant to discourse-initial expressions, which can be predictable even if they lack linguistic context at all. We propose to use script knowledge as an approximation to extralinguistic context. Since the application of script knowledge involves the generation of prediction about upcoming events, we expect that scrips can be used to manipulate the likelihood of linguistic expressions referring to these events. Previous research has shown that script-based discourse expectations modulate the likelihood of linguistic expressions, but script knowledge has often been operationalized with stimuli which were based on researchers’ intuitions and/or expensive production and norming studies. We propose to quantify the likelihood of an utterance based on the probability of the event to which it refers. This probability is calculated with event language models trained on a script knowledge corpus and modulated with probabilistic event chains extracted from the corpus. We use the DeScript corpus of script knowledge to obtain empirically founded estimates of the likelihood of an event to occur in context without having to resort to expensive pre-tests of the stimuli. We exemplify our method at a case study on the usage of nonsentential expressions (*fragments*), which shows that utterances that are predictable given script-based extralinguistic context are more likely to be reduced.

## 1 Introduction

Throughout the last 15 years, predictability effects on the choice and realization of linguistic expressions have been evidenced for a variety of languages and levels of linguistic analysis. This encompasses the findings that predictable words are articulatorily reduced [1], read faster [2] and more often pronominalized [3] and omitted [4–9]. These predictability effects have motivated the more general insight that the probability of a word to appear in context is proportional to the effort required for processing it [10, 11].

Levy (2008) [11] distinguishes two main sources that determine the probability of a word *w*_*i*_: local linguistic, i.e. intra-sentential, context, which is comprised by the *i* − 1 preceding words in the same sentence, as well as extra-sentential context, which Levy (2008:1130) subsumes under the term *CONTEXT* in Eq 1.

$$\begin{array}{c} difficulty\propto \log P(w_{i}|w_{1...i-1},CONTEXT) \end{array}$$ (1)

Many of the previous studies on predictability effects in language focus on local linguistic context alone. This restriction to intra-sentential context, and more specifically to a window of *n* − 1 words that precede a target word, has the methodological advantage that *p*(*w*_*i*_|*w*_1…*i*−1_) can be easily estimated with *n*-gram language models trained on large text corpora. *N*-grams provide a reasonable approximation to local linguistic context, in particular when the target word appears in a sentence-medial position and when the words that determine its predictability are included in the *n* − 1 preceding words. For instance, Levy & Jaeger (2007) [4] looked into the likelihood of a relative clause given the corresponding head noun, which frequently immediately precedes the relative clause and hence is contained in the context that a bigram or trigram model takes into account.

However, the likelihood of a word is also determined by linguistic material outside the *n*-gram window, that is, in a preceding sentence, or even by extralinguistic context. Effects of linguistic expressions in prior discourse can be taken into account with more recent and advanced language modeling techniques [12–18], but since these models are trained on text corpora too, they do not take into account extralinguistic context. Modeling effects of extralinguistic context is particularly important in absence of linguistic context, i.e. when an utterance appears discourse-initially, since in that case predictability effects must be triggered by extralinguistic context alone. For instance, in a situation where Ann and Bill are sharing a pizza, when Bill realizes that Ann’s plate is empty, he could ask (1). In this situation, after processing *would you like* the most likely continuation in the pizza scenario probably is *another slice*. This probability is exclusively caused by extralinguistic context, since in other contexts, like a relaxed day at the pool or a conversation about career opportunities, the other continuations in (1) seem more likely than *another slice*.

(1) Would you like *another slice / some ice cream / a new job*?

In this article, we present a method to estimate the likelihood of linguistic expressions given extralinguistic context based by using scripts [19], i.e. knowledge about the stereotypical time-course of everyday activities, as an approximation to extralinguistic context. We operationalize script knowledge with probabilistic event chains extracted from the DeScript corpus of script knowledge [20]. Since the activation of script knowledge involves the prediction of upcoming events [21–28], we expect that script-based stimuli can be used in psycholinguistic experiments in order to quantify and manipulate the likelihood of linguistic expressions. Previous work on script knowledge used mostly hand-crafted script representations, which were based on researchers’ intuitions and had to be carefully pre-tested. Since our method is based on data provided from a larger number of participants who contributed to DeScript, it provides a more reasonable approximation to the script knowledge of an average participant in an experimental study. Furthermore, it does not require additional production and/or norming studies before the main experiment which are needed in case of materials that are only based on intuitions and yields precise likelihood estimates for each event.

We apply our method to a case study on the usage of nonsentential utterances, or *fragments* [29]. In section 2 we sketch an information-theoretic account of fragment usage, which predicts that predictable utterances are more strongly reduced, so that fragments are more strongly preferred in predictive contexts. In our experiment we manipulate utterance likelihood with script-based event chains. Section 3 describes how the event chains were generated based on the DeScript corpus of script knowledge [20]. Section 4 presents an experiment on the usage of fragments, which confirms both the suitability of our method of estimating event probabilities based on script-based event chains and the predictions of our account of fragment usage. Finally, section 5 summarizes the main contributions of this article.

## 2 Predictability effects on omissions in fragments

We exemplify our method at the case of an investigation on the usage of fragments, i.e. apparently subsentential utterances like (2a) [29]. Despite their reduced form, the fragment in (2a) is interpreted as meaning-equivalent to the corresponding sentence (2b) in an appropriate context.

(2) [Passenger to taxi driver after entering the vehicle:]
  a. To the university, please.
  b. Drive to the university, please.

Fragments have been extensively discussed from a syntactic perspective, in particular with respect to syntactic aspects [29–37], but the question of when and why speakers use fragments has hardly been looked into so far. We hypothesize that the choice between a fragment and a full sentence is constrained by the tendency to reduce predictable expressions more strongly. Investigating this empirically requires an appropriate model of extralinguistic context, because the likelihood of the omitted word *drive* is determined by the extralinguistic situation: Both the driver and the passenger know that the passenger is very likely to tell the driver the intended destination after entering the vehicle. Therefore, fragments provide an appropriate testing ground for our approach to modeling extralinguistic context.

The tendency to reduce predictable expressions has been observed on different levels of linguistic analysis, ranging from phonetics [1, 38–47] and morphology [48] to the omission of predictable words [4, 6–9, 49]. If this principle applies to fragments as well, we expect that fragments are more strongly preferred over the corresponding full sentence if the omission of words that are predictable in a specific context results in a well-formed fragment.

The studies cited in the preceding paragraph analyze the omission of predictable expressions as driven by information-theoretic processing constraints. These constraints require the speaker to optimize the speech signal with respect to properties of the communicative situation and to properties of the hearer in order to increase the communicative efficiency. For the omission of entire words, this idea is captured by the Uniform Information Density (UID) hypothesis [4]. UID is based on two abstract information-theoretic concepts that stem from the model of efficient communication through a noisy channel [50]: a probabilistic notion of information and the assumption that communication occurs through a noisy channel with a limited capacity. Information, or *surprisal* [10], is defined as −log_2_ *p*(*word*|*context*), i.e. the negative logarithm of a word’s likelihood to appear in a given context. The more likely a word is, the less information it conveys. Since Hale (2001) [10], this notion of information has been related to processing effort: The more information a word conveys, the more processing effort it requires [11, 51]. Given the link between information and processing effort, we interpret channel capacity as an upper bound to the cognitive resources of the hearer. Under-utilizing channel capacity results in inefficient communication, and exceeding it results in processing difficulties. Taken together, speakers aim at adapting their utterance to the goal of communicating at a rate close to channel capacity without exceeding it. Information maxima that exceed channel capacity shall be avoided, just like information minima that do not make use of the full cognitive resources available to the hearer.

If the omission of words in fragments is also driven by UID, we expect that predictable words are more likely to be omitted, because this avoids inefficient local information minima. In predictive contexts, i.e. when an utterance referring to a specific message is more likely, the words within this utterance will be more likely on average. Therefore, the utterance will be more likely to be reduced and to appear as a fragment. In contrast, unpredictable words will more often be realized so that unpredictable utterances appear more often as full sentences.

## 3 Scripts as an approximation to extralinguistic context

The approach that we take in this article consists in using *script knowledge* [19] as an approximation to extralinguistic context. In the taxi example in (2), the material omitted in the fragment is predictable because both the driver and the passenger know that the latter will probably tell the driver the destination in the described situation. Both have knowledge about the stereotypical time-course of events involved in a taxi ride. We therefore take the predictability of an event in a script-based situation as a proxy to the likelihood of an utterance referring to it. To our knowledge, scripts have not been used for this purpose in previous research. Previous studies have shown that script knowledge modulates the predictability of linguistic expressions like single-word primes [22, 25, 26], words within complete sentences [52–54] and complete sentences within script bases stories [21]. However, in none these studies the target expression was a (spoken) utterance by one of the participants in the script-based story but described an event from the narrator’s perspective.

Before we discuss our approach in greater detail, note that there might be cases where the likelihood of an event is not correlated with the likelihood of an utterance related to this event. Specifically, when an event is extremely likely or obvious, an utterance about it might appear to be uninformative to the listener. Consider for instance the contrast in (3): Even though somebody crossing a green traffic light is probably more likely than somebody crossing a red one, the corresponding utterance in (3a) might be less likely than (3b), because it does not communicate interesting information in absence of additional inferences. See Kravtchenko & Demberg (2015) [55] for evidence that underinformative sentences actually *trigger* such inferences, which in case of (3a) could be the implicature that Peter usually does not cross green traffic lights.

(3)  a. This morning, I saw Peter crossing a green traffic light.
    b. This morning, I saw Peter crossing a red traffic light.

This observation could suggest that highly predictable utterances appear to be marked as compared to less predictable ones. The results of our study however suggest that this prediction is not borne out: Predictable utterances are preferred across the board over unpredictable ones and this holds more strongly the more knowledge about a situation subjects possess, i.e. the *more* predictable the event referred to by the utterance is to them.

Scripts are a particularly promising model of context for the study of predictability effects on omissions because they have been argued to be accessed in order to retrieve implicit material, like events that necessarily took place in the script but were not mentioned in a script-based story [19]. For instance, Bower et al. (1979) [21] showed that in a recall task subjects inferred actions that were not originally mentioned in a script-based story. Consequently, if the use of scripts involves the activation of unmentioned script events, we expect that scripts might also contribute to the recovery of material which is unarticulated in fragments. In our taxi example, if both interlocutors expect the passenger to tell the driver the destination the corresponding utterance will also be likely. This assumption is supported by the observation that nouns describing event labels prime typical participants in this event (e.g. *accident—policeman*) and a series of similar relations hold between other pieces of script-based information, like typical locations, instruments and objects [58].

The assumption that an utterance telling the driver the destination is predictable in the taxi scenario however requires not only that speakers can access some representation of a script during conversation, but also that they use this representation in order to generate inferences about *upcoming* events and linguistic expressions during language comprehension. Otherwise, any utterance that typically occurs somewhere in the taxi script might appear to be predictable once the script is active. Previous research on scripts however indicates relatively robustly that interlocutors are sensitive to the time-course of scripts and generate expectations about upcoming events. Bower et al. (1979) [21] show that subjects are aware of the typical order of script events, since they remember events in script-based stories better when they appear in their canonical position. Mc Koon & Ratcliff (1986) [22] find that subjects predict likely outcomes of actions described in stories, and similarly van der Meer et al. (2002) [25] conclude that descriptions of events that are likely to follow a script event are easier to process than those that precede this event. More recently, script-based expectations have been reported to be triggered on a more fine-grained level by switching single words within the context describing an event. Bicknell et al. (2010) [27] show that typical objects of script-based actions (e.g. *the spelling* for *the journalist checked*) are read faster and trigger a reduced N400 as compared to unpredictable ones (*the brakes*). They manipulated expectations via the subject noun phrase (*the journalist* vs. *the mechanic*), so that *the mechanic* checking something increases the likelihood of *the brakes* and *the journalist* that of *the spelling*. Matsuki et al. (2011) [54] find a similar effect in self-paced reading and eye tracking studies when the object of a verb is interchanged (e.g. *to wash a car with a hose*, *to wash one’s hair with shampoo*). The finding by Metusalem et al. (2012) [56] that words which are related to an event but unexpected in linguistic context yield a reduced N400 as compared to words that are unrelated to the event furthermore suggests that script-based predictability effects are independent from those driven by linguistic context. Even though these studies clearly demonstrate that predictability effects for linguistic expressions are driven by script-based expectations, not all of them rely on scripts in the sense of Schank & Abelson (1977) [19], i.e. knowledge about the time-course, participants and objects of *stereotypical* everyday actions. For instance, the events in van der Meer et al. (2002) [25] are described on a very fine-grained level *bite off, chew, swallow, digest* as compared to the coarser event labels in other studies.

The observation that scripts facilitate the comprehension of predictable experimental stimuli evidences that script-based expectations are relatively similar between participants. That many speakers within a community share script knowledge is a prerequisite for the application of scripts in language comprehension, since only in that case a speaker can assume that the hearer has the relevant script knowledge to process script-based utterances. Bower et al. (1979) [21] present evidence from data collected with a production task that script knowledge is indeed relatively homogeneous across participants. This is an important advantage of scripts over aspects of world knowledge that are not contained in script knowledge, since they do not necessarily trigger similar expectations across participants.

### 3.1 Probabilistic event chains as script representations

Studies investigating script knowledge in general, its effects on the comprehension of script-based stories or predictability effects driven by script knowledge with experimental methods require that materials used in the experiments are aligned with participants’ actual script knowledge. The intuitions of an individual researcher about the stereotypical time-course of e.g. cooking scrambled eggs do not necessarily match the script knowledge of an average participant in an experimental study. Even though some early studies [21, 22] did not conduct norming studies and relied mostly on intuitions, in more recent studies on script knowledge, researchers conducted norming studies preceding the main experiment to ensure that their materials actually match the participants’ script knowledge.

In some of the previous studies, stimuli were first constructed based on intuitions and then evaluated in norming studies like plausibility ratings and/or cloze tasks. Norming studies were used to assess whether that certain events, objects or participants were more predictable in the context of script-based stories than alternative events, objects or participants [25, 27, 28, 53, 56]. Other researchers used production and cloze tasks not (only) to verify their intuitions, but to actually determine the most predictable expression in a specific context [26, 52, 54, 57, 58]. Bower et al. (1979) [21] even collected data about the complete time-course of scripts for a reduced number of scripts with a production task, but not all of their experiments rely on these data.

Conducting norming studies increases the cost and effort required to prepare and evaluate the study, and since the resulting data, such as cloze probabilities, are collected for highly specific stimuli, even minimal modifications to the materials require further norming studies. Furthermore, cloze tasks provide precise probability estimates for individual words within a sentence, but they do not allow for the estimation of the probability of the utterance as a whole. Our study however investigates the hypothesis that overall more predictable utterances are more likely to be reduced, therefore we need an estimate of the overall likelihood of an utterance rather than an estimate of the likelihood of individual words given the initial part of a sentence. Lemke et al. (2020) [59] used a free production task to quantify utterance probabilities given extralinguistic context, but this approach requires a large amount of pre-processing, since participants’ responses are relatively diverse as compared to responses in a cloze task, which are constrained by the intrasentential context. This being said, depending on the research question, cloze norming studies might be more adequate to estimate some script-driven predictability effects, specifically when it comes to more fine-grained probability differences on the word level given some fixed intrasentential context. For instance, when semantically relatively similar high and low clause probability words are compared (see e.g. [53]), a norming study appears to be more appropriate because it yields more precise probability estimates for a particular word. In turn, our corpus-based approach focuses on the likelihood of events, no matter how this event is eventually lexicalized. This is specifically relevant in case of fragments, where the hearer must infer material that is left implicit. For instance, in the taxi scenario, the passenger’s fragment *to the university* will be understood no matter whether the driver reconstructs the missing words as *bring me …*, *I need to go …* or *drive …*, if he assigns the structure such a precise representation at all.

**Fig 1:**
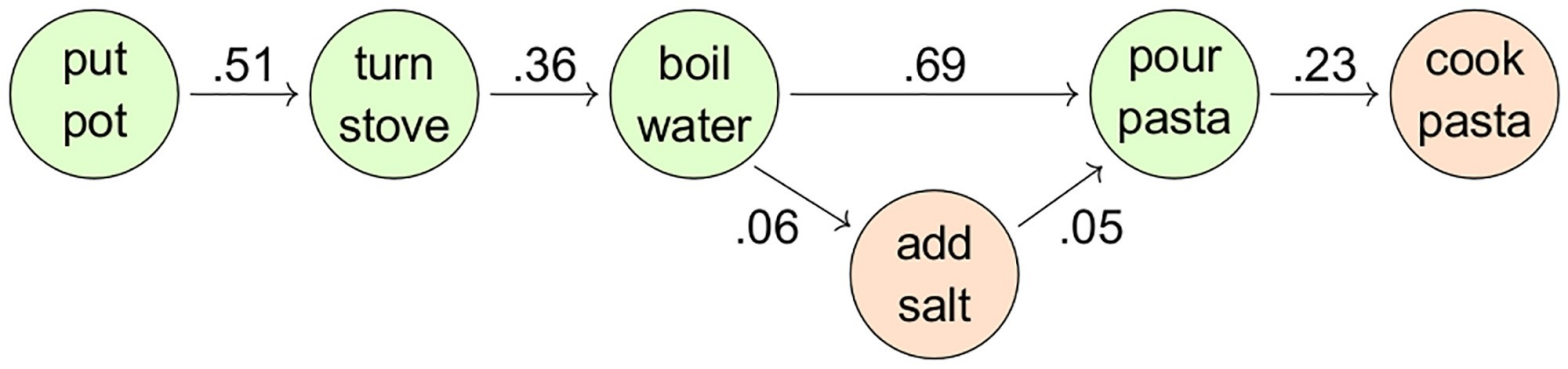
Sample event chain with transition probabilities between events estimated from the preprocessed DeScript data. The four events marked in green were used in the item for the pasta scenario.

In our study, we rely on DeScript [20], a publicly available crowd-sourced corpus of script knowledge in order to model script knowledge. We construct our stimuli based on probabilistic event chains (see Fig 1 for a sample) of the most likely events to follow each other in the corpus and use these event chains to quantify the likelihood of a target event. We represent scripts as probabilistic networks rather than as mostly linear sequences of events [19]. In such a network, each event is assigned a state *e*_*i*_ which has a certain transition probability to another state *e*_*j*_. The transition probability *p*(*e_j_*|*e_i_*) is equivalent to the likelihood of *e*_*j*_ to follow *e*_*i*_ and can be estimated for each pair of states 〈*e*_*i*_, *e*_*j*_〉 in the total set of states that is determined by the script. Transition probabilities can range from 0, i.e. *e*_*j*_ never follows *e*_*i*_, to 1 in case *e*_*j*_ always follows *e*_*i*_. Based on these transition probabilities we extract a linear sequence of the most likely events to follow each other, like the event chain in Fig 1, as the structure underlying our materials.

### 3.2 Preprocessing DeScript

#### 3.2.1 Corpus and script selection

DeScript [20] is a crowd-sourced corpus of script knowledge that has been collected on Amazon Mechanical Turk and that comprises about 100 event-sequence descriptions (ESDs) by native speakers of English for each of 40 scripts. The corpus is freely available and provided in an XML format. The scripts contained in the corpus differ both in their internal complexity (e.g. *washing dishes* and *going to a funeral*), that is, variation between (the number of) events and their ordering, and with respect to the number of participants involved. An ESD consists of a series of events provided by an individual subject that describes how the script typically develops. (4) and (5) are two sample ESDs from the cooking pasta scenario.

(4) a. Look up a pasta recipe
   b. Get all the sauce ingredients
   c. Boil the noodles until well done
   d. Cook the sauce
   e. Combine the sauce and the pasta

(5) a. Put water in a pot
   b. Turn the stove on
   c. Put the pot on the stove
   d. Boil water
   e. Put pasta in the water
   f. Wait for pasta to cook

Since the goal of our approach is to estimate the probability of an event, such as *pouring the pasta into the boiling water*, given the previous events in the script, the examples in (4) and (5) already illustrate some issues that we have to address. First of all, a single event can be described in different ways, as the roughly meaning-equivalent (4c) and (5f) show. Therefore, it is necessary to assign each event an individual label before estimating its probability, otherwise the probability of the event would be split among different lexicalizations. In addition to the ESDs, DeScript contains gold standard paraphrases for 10 of the scenarios, which unify these varied descriptions to a set of mutually exclusive event labels that determine which lexicalizations can be subsumed under a label like choose recipe. However, these paraphrases are available only for a subset of 10 scenarios. Therefore, instead of using the gold standard paraphrases, we pre-processed a larger part of the corpus in order to obtain such unique event labels. Second, ESD for a single script differ in granularity, explicitness and the events that participants include in the script. In part, this probably reflects genuine differences in participants’ script representations, such as the inclusion of looking up a recipe in (4) but not (5). However, there are also instances of events which must occur in the script, but which are sometimes left implicit by participants. For instance, the ESD (4) does not mention the event of turning the stove on, even though this event must necessarily have occurred since the water could not boil otherwise. In section 3.2.3, we discuss in greater detail how we addressed these issues.

Since our experiment investigated encoding preferences on the form of linguistic utterances in communicative settings, like the choice between the sentential and the fragment utterance in the taxi scenario in (2), it was necessary that (at least) two participants are present in the script. However, only 17 of the scripts in DeScript contain two participants that talk to each other, such as the customer and the operator in a pizza ordering script. The remaining scenarios (e.g. *cooking pasta*, *making scrambled eggs*) consist in series of events for which no second participant is specified. In order to construct 24 script-based stimuli for our experiment, in addition to the scripts that involved two participants in DeScript, we selected some scripts for which it is reasonable to assume that the time-course of the script is not affected by the introduction of such an additional participant. For instance, in the pasta cooking scenario it is plausible that two friends prepare a meal together and chat in the meantime. In our statistical analysis we included a ScriptType predictor in order to account for potential differences between scripts which originally contained two participants and those for which we introduced a second participant. Such a difference would have been expected given the distinction between *situational* and *instrumental* scripts in [19], who attribute a reduced predictive potential to the latter [19, 66]. However, our statistical analysis did not reveal any significant difference between both script types.

#### 3.2.2 Generation of event labels

**Fig 2:**
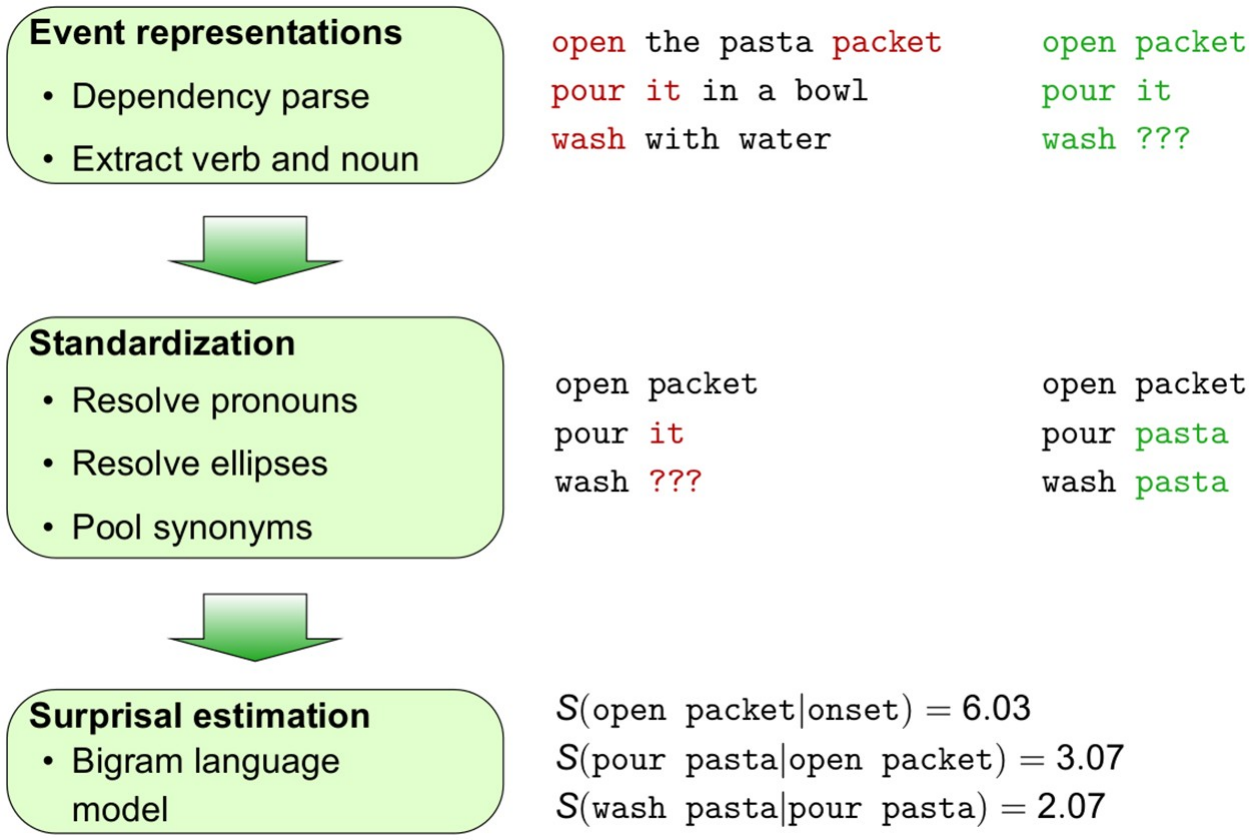
Overview of the preprocessing procedure applied to DeScript for a sample sequence of events from DeScript. First, verb-noun pairs were extracted as event representations. The data were then standardized by resolving pronouns and ellipsis and pooling synonym words, so that each event was assigned a unique label. Finally, surprisal was estimated with a bigram language model.

Estimating the likelihood of an event in context of the preceding one(s) requires to transform the event descriptions contained in the corpus into event sequences. The likelihood of an event can then be estimated with *n*-gram language models. For this purpose, each event in the data for a script must be assigned a unique label that distinguishes it from other events. In what follows we describe how we preprocessed the DeScript data in order to generate event chains that underlie our stimuli. Fig 2 summarizes this preprocessing procedure.

Following Manshadi et al. (2008) [60], each event label consisted in the main verb of the corresponding sentence and the post-verbal noun, which is its direct object in case of transitives. This method transforms the original production data, which consist of (frequently elliptical) sentences to event sequences like (6). Note that the only purpose of the labels is to distinguish between events during language modeling, and that it does not matter whether e.g. turn stove is the most accurate description of the event of turning the stove on.

(6) put pot  turn stove  boil water  pour pasta

**Fig 3:**
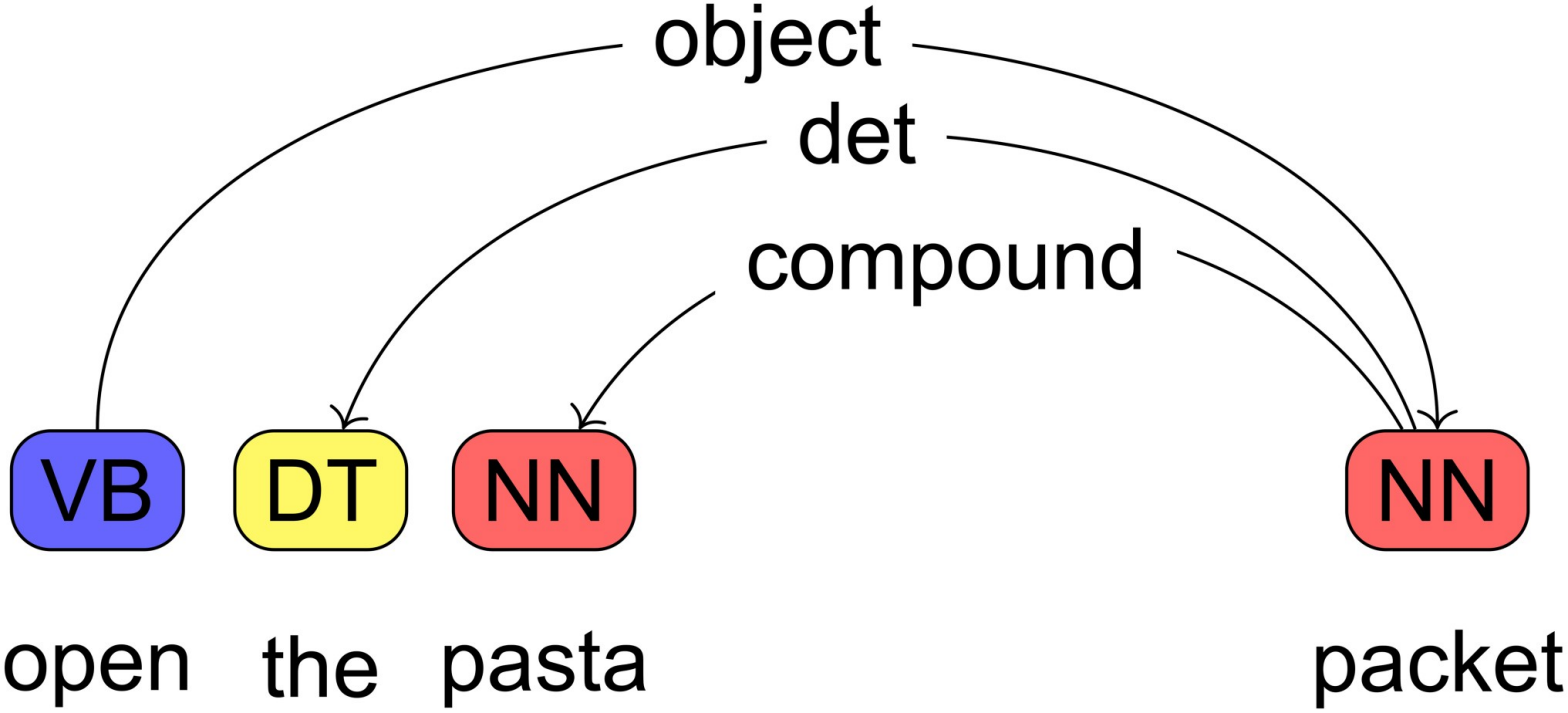
POS tags and dependencies for a sample event description. POS tags are given in the colored boxed, dependencies on the arrow labels. Based on these representations we extracted the verb and its object noun, and manually reviewed the data in case the dependency parser did not find an object noun.

We generated event labels by extracting the main verb and the noun from the event descriptions in DeScript. The raw DeScript data were Part of Speech-tagged with the Stanford parser [61] for English contained in the Python Natural Language Toolkit (NLTK) [62]. The data were then dependency-parsed with the Stanford dependency parser contained in the NLTK. Fig 3 provides an overview of the outcome of POS tagging and dependency parsing for a sample event description. The parser was often misguided by the high ratio of elliptical event descriptions, subject omissions and verb-first imperatives that are infrequent in the written corpora on which it was trained. In such situations it interprets e.g. initial verbs as nouns, specifically when there are homonym with nouns like *set* and then assigns wrong POS tags to following words. We addressed this issue by using a version of the parser that had been trained on a modified set of training corpora by Michaela Regneri (see Regneri (2013) [49, 50, 63] for details) from which some of the sentence-initial noun phrases had been removed. This method allows the parser to analyze English SVO structures with missing subjects as such instead of analyzing initial verbs as nouns and hence results in a higher accuracy of the parser.

After parsing, the main verb and its direct object were extracted from the parsed data. In case there was no direct object, either because the verb was intransitive, the object had been omitted or left implicit, or no direct object was recognized due to tagging and/or parsing errors, a placeholder was inserted and reviewed manually. This procedure required the resolution of pronouns and ellipsis as well as the correction of parsing or tagging errors, since otherwise event descriptions like *boil the pasta for 8 minutes*, *boil them for 8 minutes* and *boil for 8 minutes* would be treated as referring to different events by the language modeling software that we used for probability estimation.

#### 3.2.3 Preprocessing of event chains

The resulting verb noun event representations for each scenario were manually preprocessed in order to pool both synonym words and syntactically differing descriptions of the same event. The goal of this procedure was to assign each event a unique label, because otherwise the language model would treat instances of the same events as distinct events. For instance, in the scrambled eggs scenario, the event of pouring the eggs into the pan was described with all of the sentences in (7). Without manual preprocessing, 7(a) would be represented as pour egg, 7(b) as put content and so on.

(7) a. Pour eggs into the pan
   b. Put contents of bowl in pan
   c. Pour them into a pan
   d. Pour in pan

This variation within the event descriptions in DeScript requires the assimilation of the individual descriptions, so that a single verb noun label is assigned to each individual event. The rationale for this procedure was that each script should involve a closed class of mutually exclusive participants (both animate and inanimate, i.e. roles and props in the terminology in Schank & Abelson (1977) [19]) and that there should only be one label for each participant. Similarly, there should be a unique label for each event within the script. The first requirement ensured that synonyms, such as *pan* and *skillet*, were pooled to a single lemma, and the second one ensured that different descriptions of the same action, like those given in (7), were assigned the same label. This is crucial for interpreting the language models trained on the event sequences because otherwise the probability mass of e.g. the event referring to pouring the eggs into the pan would be split among the events pour egg, put content and pour in. In order to obtain unique labels for each event, we also had to resolve ellipses and the reference of pronouns. Finally, the data for each scenario were screened using an R script in order to ensure the uniqueness of each participant, each action, and consequently each event within the script.

### 3.3 Event surprisal estimation

After assigning each event a unique label, we estimated its conditional probability with bigram language models trained with the SRILM tool kit [64]. Unlike in the case of *n*-gram models trained on text corpora, the primitive expressions are not words, but event labels, and the models return the probability of an event given the previous one. The usage of higher order *n*-grams would not have been reasonable given about 100 event chains per scenario. Even after preprocessing, relatively homogeneous scenarios such as the train ride script had a vocabulary size (i.e. number of different primitive events) of 121, more diverse scenarios, such as e.g. making scrambled eggs, even had a unigram vocabulary size of 192. As there is often more than one possible successor for each event, this yields a vocabulary of 351 bigrams for the train and of 672 bigrams for the eggs scenario.

Preprocessing the DeScript data for 24 scripts using both automatized and manual procedures yields a data set that we used to estimate the likelihood of a script-based event given the previous one. The method described in this section ensures that the probability mass of an event is not split among alternative lexicalizations, and that speakers’ script knowledge is a probabilistic estimate of how people in a determinate community represent a given script, including potential differences in the events involved as well as in their ordering and granularity. The event surprisal estimates for the 24 scenarios are available under http://hdl.handle.net/21.11119/0000-0007-E18F-A.

## 4 Acceptability rating experiment

### 4.1 Approach

We used an acceptability rating study to test whether fragments are more strongly preferred in predictive contexts, as our information-theoretic account predicts. From a methodological perspective, this will also show whether corpus-based event chains allow for controlling and manipulating expectations based on extralinguistic context. Since the event chains are based on a relatively large and publicly available data set, this method would could reduce the need for pre-testing of hand-crafted stimuli or production tasks preceding the creation of experimental material.

We compare the acceptability of predictable and unpredictable DP fragments (8a,b) to that of corresponding full sentences (8c,d) in a 2 × 2 (Predictability × Sententiality) rating study in German. Our account predicts an interaction between both factors, i.e. that fragments are preferred specifically in the predictable condition. If the predictability manipulation works, we also expect a main effect of Predictability, i.e. that utterances that refer to likely script events are overall perceived as more natural.

(8) Annika and Jenny want to cook pasta. Annika put a pot with water on the stove. Then she turned the stove on. After a few minutes, the water started to boil. Now Annika says to Jenny:
  a.  Die Nudeln, bitte.
      the pasta  please              Predictable
      The pasta, please.
  b.  Den  Küchentisch, bitte.
      the.acc kitchen.table please
      The kitchen table, please.          Unpredictable
  c.  Schütte die Nudeln ins  Wasser, bitte.
      pour the  pasta in.the water please        Predictable
      Pour the pasta into the water, please.
  d.  Deck schon mal den  Küchentisch, bitte.
      set already prt the.acc kitchen.table please   Unpredictable
      Set the kitchen table, please.

### 4.2 Materials

All materials consisted of a script-based context story involving two participants, which was followed by a target utterance produced by one of these participants. Each context story had four sentences, the first of which introduced to the script and mentioned its title, e.g. *cook pasta*. The remaining three sentences refer to events which are most likely to follow each other given the bigram event probabilities in DeScript. The context story ends with a sentence like *now Annika says to Jenny* that determines which of the participants produces the target utterance.

The story ensures that the event referred to by the target sentence in the predictable condition (9a,c) (pour pasta) is the most likely event to follow the previous one (boil water). The other two events in the context story are selected by the same criterion, i.e. so that the event that follows them in the story is the most likely one to do so in the script representations derived from DeScript. The target event had a mean bigram surprisal of 2.13 bits (which is equivalent to a likelihood of 22.8%). The average bigram surprisal of all three events in the event chains was 2.18 bits (22.1%). Events that were overall rare (*n* < 8) in the script data were not considered in this process. Otherwise, for instance an event that was mentioned by only one out of 100 participants would be taken to represent the script knowledge of the complete population. In the unpredictable conditions, the target utterance refers to an event that did either not appear in the script data at all, or that has a probability of 0 in this context, but that seemed intuitively plausible to be talked about in this situation. All sentential target utterances had a transitive main verb, whose direct object DP was used as the target utterance in the fragment conditions. We added a *please* to all materials of a token set whenever this made the utterances sound more natural.

### 4.3 Procedure

The experiment was run over the Internet using the LimeSurvey survey presentation software. 48 self-reported native speakers of German were recruited on the clickworker.com crowdsourcing platform and were rewarded with ⋹4.00 for participation. The experiment was conducted with the approval of the ethics committee of the Deutsche Gesellschaft für Sprachwissenschaft (German Society for Language Science). All subjects gave informed consent and were compensated with monetary payment for their participation. Subjects were asked to rate the naturalness of the target sentence, which was presented in italics, in the context of the context story on a 7-point Likert scale (7 = fully natural). They were assigned to one of four lists, to which materials were allocated by a 2 × 2 Latin square design, so each subject saw each of the 24 token sets once and 6 items in each of the four conditions. Materials were mixed with 21 items from an unrelated experiment and 44 unrelated fillers. Both the fillers and the materials of the unrelated experiment resembled the items in having a context story and an italicized target utterance which subjects rated. In the materials of the other experiment and in 18 out of the 44 fillers the target utterance was a fragment, in the remaining 26 fillers it was a sentence. This ensured that sententiality was almost balanced throughout the experiment. Materials were presented in individual pseudo-randomized order, so no two items or fillers of the same category immediately followed each other. Three subjects who rated more than 2 out of 5 ungrammatical controls as natural (6 or 7 points) were excluded from further analysis.

The main experiment was followed by a script familiarity questionnaire in order to assess the participants’ knowledge about the scripts underlying our materials. The motivation for including this questionnaire was that our script-based manipulation of the likelihood of utterances and expressions requires that participants possess the relevant script knowledge. For instance, in the case of the pasta cooking scenario described above, only participants who know how to cook pasta will expect that the pasta are to be put into the pot when the water starts boiling. Subjects who do not know the stereotypical time-course of a script will not be able to predict the likely target event in the predictable condition from the context story. The participants’ script familiarity might affect the acceptability rating data in two ways. First, there could be individual differences between participants with respect to their knowledge about a particular script. Second, there might be overall differences in familiarity between scripts. Some of the scripts in DeScript describe situations about which almost every German subject will have knowledge, such as grocery shopping or cooking pasta, but this may not be the case to the same extent for scripts like fixing a bicycle tire or going to the sauna. We expect that participants perceive the predictable conditions as more natural the more familiar they are with a script and consequently that the preference for the predictable conditions is stronger the higher the familiarity with a script is across participants. In the questionnaire, subjects were asked to check on a 5-point scale how familiar they were with each of the scripts underlying our materials (5 = very familiar). In the instructions, we defined familiarity as “knowing how these situations typically develop”. We asked subjects to consider not only their own experiences, but also knowledge reported by others or gained through the media. The scenarios were described by nonsentential phrases, such as “train ride” or “baking a cake” equivalent to the script titles. The z-transformed script knowledge scores were used as a predictor in the statistical analysis. Due to a technical problem, only the script knowledge scores for 22 out of the 24 scenarios were recorded. Since our statistical modeling approach is robust to missing data, it allows us to include ScriptFamiliarity as a predictor in our analysis despite this issue. If the acceptability of fragments is conditional on script knowledge, the expected contrast between predictable and unpredictable utterances should increase the more familiar subjects are with the scenario. From the information-theoretic perspective, we might also expect a three-way interaction of ScriptFamiliarity, Predictability and Sententiality, because participants who lack script knowledge fail to reconstruct the otherwise predictable material that is omitted in predictable fragments.

### 4.4 Results

**Fig 4:**
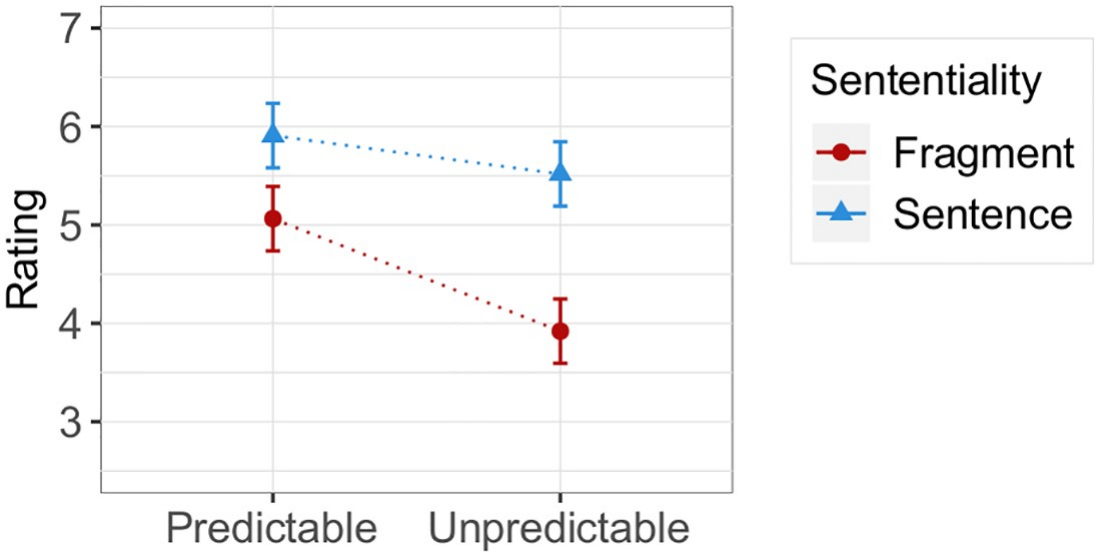
Mean ratings + 95% CIs across conditions.

Fig 4 shows the average ratings across conditions. We analyzed the data with Cumulative Link Mixed Models [65], starting with a full model that includes main effects for all predictors and all two-way interactions between them. Categorial predictors (Predictability, Sententiality, ScriptType) were sum-coded as (-1,1). We then used a backward model selection procedure and successively excluded the fixed effects from the model that did not significantly improve model fit, as evidenced by likelihood ratio tests. The full model contained main effects of Sententiality, Predictability, ScriptType (whether the DeScript data originally contained two participants or whether the script was adapted for our materials), the Position of the item in the experiment, and the z-transformed ScriptFamiliarity score from the script knowledge questionnaire that followed the main experiment. Besides all two-way interactions, we also included the Sententiality:Predictability:ScriptFamiliarity three-way interaction, which would show whether the Sententiality:Predictability interaction predicted by information theory is stronger the more familiar subjects are with the scenario. The model contained by-item random intercepts and slopes for Sententiality, Predictability and ScriptFamiliarity and by-subject random intercepts and slopes for these predictors and all interactions between them, including the three-way interaction.

The final model (see Table 1) has the same random effects structure as the full model and contains significant main effects for both experimentally manipulated predictors: The main effect of Sententiality (*χ*^2^ = 30.5, *p* < 0.001) reveals a general preference for sentences over fragments, and the main effect of Predictability (*χ*^2^ = 10.49, *p* < 0.01) shows that predictable utterances are also overall preferred. The significant interaction (*χ*^2^ = 9.61, *p* < 0.01) between both predictors indicates that, as we expected, the preference for sentences is weaker in the predictable condition: Fragments are more acceptable when they refer to a predictable event.

**Table 1:** Fixed effects in the final CLMM.

Predictor	Estimate	SE	*χ*^2^	p-value
Sententiality	-0.958	0.143	30.5	<0.001***
Predictability	-0.554	0.162	10.49	<0.01**
ScriptFamiliarity	-0.012	0.109	0.03	0.86
Position	-0.021	0.002	21.57	<0.001***
Sententiality: Predictability	-0.22	0.073	-9.61	<0.01**
Predictability: ScriptFamiliarity	-0.206	0.094	5.08	<0.05*

**Fig 5:**
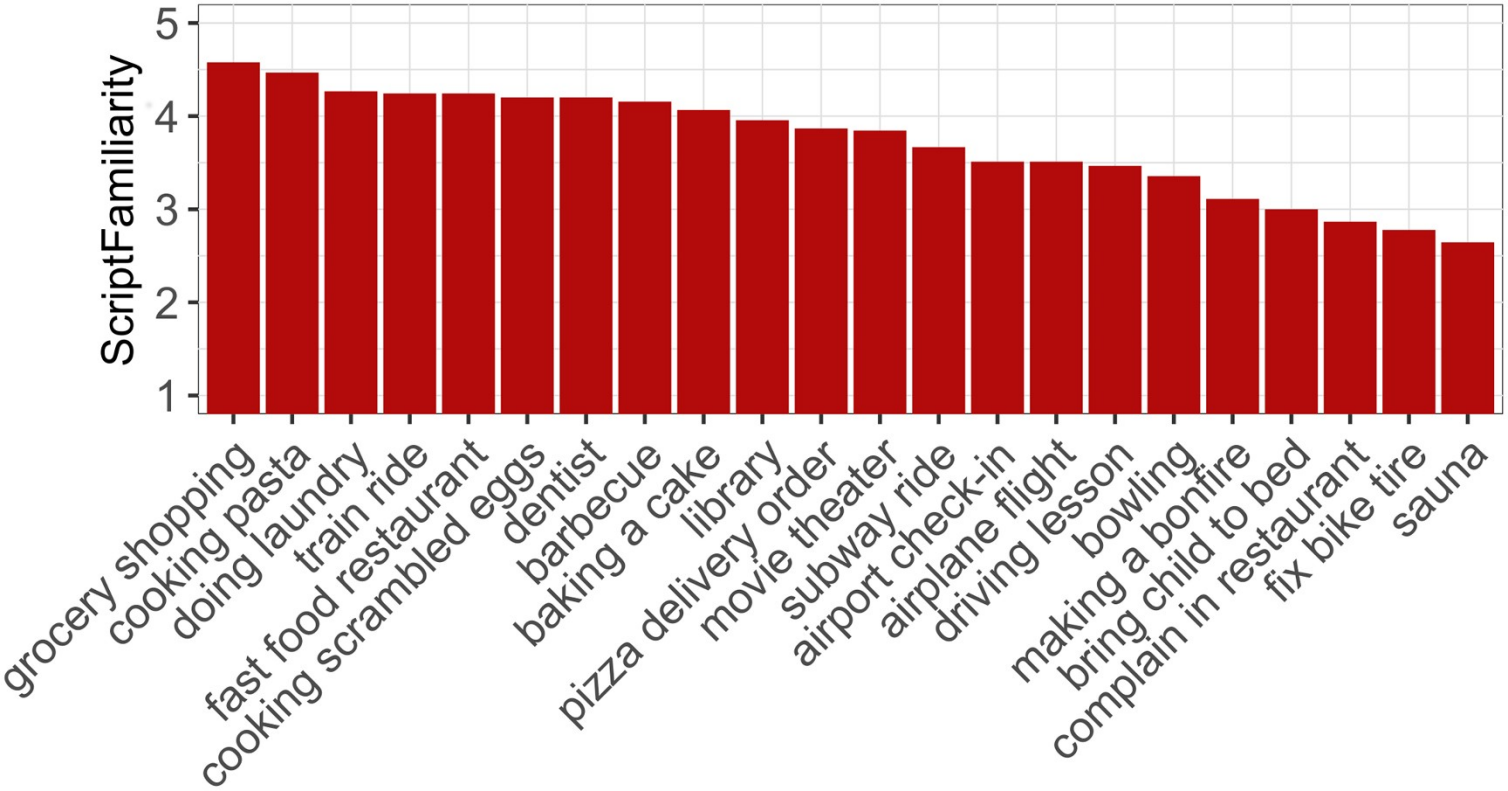
Mean ScriptFamiliarity score for the scripts tested in the experiment.

As Fig 5 shows, there was some variation in ScriptFamiliarity between scenarios, even though the absolute ratings suggest that all of them seem to be relatively familiar to the participants. Our model shows that ScriptFamiliarity (*χ*^2^ = 0.03, *p* > 0.8) has no significant main effect on the ratings, but it significantly interacts with Predictability (*χ*^2^ = 5.08, *p* < 0.05): The more people know about a scenario, the more they distinguish between the predictable and unpredictable conditions. Our analysis provides no evidence that this interaction is further conditioned by the degree to which subjects possess the relevant script knowledge since the three-way interaction Sententiality:Predictability:ScriptFamiliarity is not significant (*χ*^2^ = 2.36, *p* > 0.1). An anonymous reviewer to PLOS ONE pointed out that the three-way interaction might be non-significant due to the relatively low number of participants. This could be addressed with the replication of the experiment with a larger sample size. Since the non-significance of the three-way interaction is not central to our predictions, we leave this issue open for future research.

The absence of any significant effect of ScriptType or interaction with other predictors suggests that there is no difference between the adapted scripts and those that contained two participants in the DeScript data. Finally, there is a theoretically uninteresting Position main effect that however does not interact with any of the other predictors and shows that ratings improve in the course of the experiment.

### 4.5 Discussion

Our experiment had two main goals: From a methodological perspective, we tested the suitability of script-based event chains as an approximation to extralinguistic context. From a theoretical viewpoint, we investigated whether the usage of fragments is constrained by predictions derived from this model of context.

#### 4.5.1 Script-based event chains as an approximation to context

Our experiment suggests that script-based stories based on probabilistic event chains extracted from a corpus of script knowledge provide a reasonable approximation to extralinguistic context. The significant main effect of Predictability in the analysis of the rating data shows that utterances that refer to events that are more likely given the DeScript data are perceived as more natural by subjects. Furthermore, the interaction between Predictability and the ScriptFamiliarity scores, which were collected with the questionnaire following the main experiment, indicates that this effect is particularly strong when subjects are familiar with a scenario. Both of these findings indicate that our script-based materials allow for estimating and manipualting the predictability of utterances.

#### 4.5.2 Evidence for predictability effects on the usage of fragments

In addition to confirming the suitability of script-based event chains as an approximation to extralinguistic context, our study supports the information-theoretic prediction that more predictable utterances are more likely to be reduced. The Sententiality:Predictability interaction suggests that fragments are more acceptable when they encode a predictable message than when they encode an unpredictable one. We thus provide first empirical evidence that the perceived acceptability of a fragment and the corresponding sentence depends on the predictability of the message they encode.

It might seem surprising that sentences were rated as more acceptable than fragments even in the predictable condition. Fragments are relatively frequent in corpora [66], and if, as we hypothesize, information-theoretic constraints determine the choice of an encoding, there must be situations where a fragment is the most well-formed encoding for a message. However, information-theoretic constraints do not neglect the possibility that other factors constrain the usage of fragments as well. For instance, the written presentation modality or politeness considerations (fragments might be considered less polite, as has been argued for subject omissions [67]) might have increased the overall acceptability of complete sentences. Furthermore, not all of the fragments that we tested in our study might be the most optimal ones with respect to UID. A single sentence like (9) can serve as input for a larger set of fragments, like e.g. (10). In the pasta scenario, the PP in (10b) might actually be more well-formed than the DP in (10a): If *the pasta* is more predictable than *into the pot*, omitting the former and realizing the latter phrase conforms better to UID.

(9) Pour the pasta into the pot, please!
(10) a. The pasta!
     b. Into the pot!

Since our approach only measures the likelihood of the utterance as a whole, determining whether this explanation is correct requires being able to estimate the likelihood of individual words in fragments. Lemke et al. (2020) [59] address this issue with a data set collected in a production task.

Taken together, our experiment shows that fragments are more acceptable when they refer to a message that is predictable in context. This supports our hypothesis that predictable utterances are more likely to be reduced. The overall preference for sentences is unexpected but does not contradict the predictions of information theory. This extends evidence for UID effects on omissions in two ways. First, we find that omissions are also driven by predictability manipulations through extralinguistic context, whereas previous research investigated mostly effects of local linguistic context, i.e. *n*-gram surprisal. Second, previous studies focused on semantically relatively vacuous function words, like relative pronouns and complementizers. We find that UID also constrains omission of content words.

## 5 Conclusion

In this article we presented and evaluated a new approach to estimating and manipulating the likelihood of utterances given extralinguistic context. We used the likelihood of an event given script corpus data as an approximation to the likelihood of a corresponding utterance. Our experiment confirmed the validity of this approach at a case study on the usage of fragments: Subjects perceive utterances that refer to likely events as more natural. The observation that this effect is stronger for subjects who have more script knowledge also supports our approach, since we expect that scripts trigger expectations only in subjects who possess the relevant script knowledge. We also find that our predictability manipulation through script knowledge affects the perceived well-formedness of fragments: Like our information-theoretic account predicts, fragments are more acceptable when they appear in predictive contexts.

Our script corpus-based approach has two advantages over hand-crafted and pre-tested materials or those based on production tasks, which were frequently used in previous studies on effects of script knowledge on language comprehension. First, the data provided by a large number of workers recruited on crowd-sourcing platforms are a relatively solid approximation to the script knowledge of an average participant as compared to the researcher’s intuitions or those of a smaller sample of subjects who participate in a norming study. Second, constructed materials that have to be tested in a norming study or stimuli based on production data are often designed for a specific experiment. Instead, event chains based on a freely available corpus like DeScript provide event probabilities based on script representation provided by a relatively large number of contributors to the corpus. These probabilities can be applied in research on any kind of discourse expectations based on extralinguistic context, like our case study on fragments, without the need of conducting expensive norming studies for each individual experiment.

## Supporting information

S1 Table Acceptability rating data. (CSV)
